# Shall we walk? A user study of an app tracking time in nature in everyday life

**DOI:** 10.1177/20552076261470171

**Published:** 2026-08-12

**Authors:** Sigbjørn Litleskare, Rossano Schifanella, Giovanna Calogiuri

**Affiliations:** 1 Centre for Health and Technology, Department of Nursing and Health Sciences, 177041 University of South-Eastern Norway, Drammen, Norway; 2 Department of Public Health and Sport Sciences, Faculty of Social and Health Sciences, 3207 University of Inland Norway, Elverum, Norway; 3 Computer Science Department, 9314 University of Turin, Turin, Italy; 4 18455 ISI Foundation, Turin, Italy

**Keywords:** digital nature, smartphone application, digital health, preventive medicine, health promotion

## Abstract

**Objective:**

Little is known about how smartphone apps can encourage nature visits, and previous research suggests that human perceptions are key to both app development and understanding the quality of natural environments. This study developed and tested an app feature that tracks time in nature by calculating a green index to estimate the amount of nature at a location using map data and estimated levels of traffic noise.

**Methods:**

A formative evaluation was conducted in which 29 participants visited five pre-selected locations representing various types of urban nature, and provided ratings of “perceived nature”, perceived environmental restorativeness, and overall feeling. Additional measures included age, gender, baseline nature connectedness, perceived weather conditions, and an unstructured interview.

**Results:**

The green index vs perceived nature ratings (mean ± SD) at each location was 88 vs 49.9 ± 22.9 at location 1 (Pedestrian bridge); 63 vs 61.8 ± 15.7 at location 2 (Park); 31 vs 63.5 ± 18.7 at location 3 (River, green); 21 vs 44.8 ± 22.4 at location 4 (Trafficked bridge); and 50 vs 48.0 ± 21.4 at location 5 (River, grey), reflecting both underestimation and overestimation of the green index, with significant inter-individual differences. Perceived environmental restorativeness and feeling were positively correlated with perceived nature ratings. Qualitative data revealed varying preferences for green vs blue environments and the extent to which traffic noise influenced their perceptions.

**Conclusion:**

The divergence between app-based and user assessments suggests that such assessments should be calibrated using individualized user feedback, and that careful consideration is needed regarding the level of statistical detail presented to users.

## Introduction

Health and fitness apps have been proposed as powerful tools to promote healthy lifestyles and contribute to achieving public health goals,^1^ such as increasing the levels of physical activity.^2^ Some apps also aim to promote nature-based activities,^3,4^ but this is a largely overlooked area of health promotion and a field lacking in evidence-based approaches. Natural environments have been increasingly recognized as beneficial for people’s health through various pathways, including psychological restoration, promotion of physical activity, socialization, and buffering from various stressors such as excessive heat and noise.^5,6^ As little as 10 minutes in contact with nature can lead to acute improvements of various indicators of psychological and physiological health.^7^ For more stable health benefits, regular exposure to nature is recommended. For instance, a study by White et al. (2019) found that, compared to no nature contact in the previous week, the likelihood of reporting good health or high levels of well-being became significantly greater when participants reported a total duration of nature contact of 120 minutes per week or more.^8^ The health impact of 120 minutes of nature contact was comparable to living in a high versus low deprivation area, being employed in a high versus low social grade occupation, or achieving the recommended levels of physical activity, which signifies the importance of nature contact for public health.^8^ While the health benefits of spending time in nature are comparable to physical activity,^8^ the literature also indicates that physical activity in natural settings can provide greater benefits than physical activity in other settings, especially with respect to enjoyment and other psychological outcomes that can support an active lifestyle.^9–11^ Systematic reviews of evidence have demonstrated that physical activity in natural environments elicits more positive effects on affect, enjoyment, anxiety, and depression compared to indoor and urban environments.^10,11^ Additionally, physical activity in natural environments has been linked to increased levels of physical activity and exercise adherence,^6,9^ which highlights the potential of this type of exercise to not only induce acute benefits, but also long-term benefits due to increased levels of physical activity and nature exposure, such as enhanced cardiovascular health and reduced mortality.^5,6^ Despite clear evidence that even short periods in nature produce psychological and physiological benefits, people are spending less time outdoors.^12,13^ For example, in the UK fewer than 40% of people visit natural environments in a typical week.^14^ These trends have motivated the design of digital interventions to increase nature contact,,^15^ including smartphone apps that estimate and track time spent in natural settings.^3^

This article presents the development process of a digital feature designed to measure and provide feedback regarding time spent in nature, with the working title Sustainability and Health Motivator (SHM). SHM was developed as a feature of *Kobla*, an app that aims to promote active transport (https://kobla.no/en/). Within the project Drammen – Net Zero 2030, part of the NetZeroCities – Pilot Cities Programme,^16^ the app was expanded to promote time spent in nature and foster healthy, environmentally friendly behaviors. In the development process, we defined “nature” as health-promoting natural environments, given the app’s overarching goal of promoting healthy and sustainable behaviors.

The app and the SHM feature aim to address individual needs, in contrast to existing research that largely relies on average values for larger population groups.^8^ This personalized approach represents a central challenge in the development, as the health effects of nature exposure may vary between individuals. Previous research has shown that several individual factors may influence the benefits of nature exposure, including baseline levels of connectedness to nature,^17,18^ the type of motivation for visiting natural environments,^19^ ethnicity/culture,^8,20^ susceptibility to stress,^21^ and the level of attention directed towards natural elements in an area.^22^ The level of individuality creates difficulties when designing an app intended for a wide range of users. This is further complicated by the fact that people rate natural environments differently^23^ and that receiving feedback that is not perceived as credible (e.g., an app’s nature evaluation not aligning with your own) may be detrimental to motivation for performing a specific task,^24^ e.g., spending time in nature.

### Related work

Nature discovery apps designed to promote curiosity for nature, such as *NatureCollection* have shown that app-based exploration of nature can increase the amount of nature-based activities among children and youth.^4,25,26^ Studies based on data from global positioning systems (GPS) have also shown that GPS data can reliably estimate a person’s nature exposure for a specific time period (days, weeks), and higher GPS-measured exposure has been associated with better mood.^27^ GPS data have also revealed that most day-to-day greenspace visits are incidental, i.e., travelling through rather than to greenspace.^28^ Building on these findings, the app *NatureDose*™ combines GPS data with targeted feedback to increase the intentional levels of nature exposure in the population.^3^ The app combines GPS data with targeted feedback to increase intentional levels of nature exposure in the population (Browning et al., 2024). The app integrates smartphone sensors, geolocation data, and a multidimensional environmental dataset to estimate nature exposure at the user’s location and to assign a corresponding nature score.^3^ The assigned nature score for a given location is based on the estimated amount of nature in the surroundings and is presented to the user in five levels of nature (nature deficient, nature light, nature adequate, nature rich, nature utopia; https://www.naturequant.com/naturescore/). The specific spatial implementation and how data are integrated are not fully disclosed, nor how well the apps estimates align with user perceptions. The SHM feature aims to address this by evaluating the level of nature exposure in a person’s immediate surroundings and incorporating human perceptions of natural environments into its development. Human raters are known to capture nuanced perceptual dimensions such as aesthetic appeal, sense of serenity, and perceived biodiversity (e.g., perceived environmental quality indices). It has long been recommended to include human ratings of environments to understand their ecological quality^29^ and previous research have successfully incorporated human evaluations for apps designed to promote physical activity.^2^ Human raters provide further insight into psychological restoration and health‐related attributes of green and blue spaces that purely algorithmic approaches may overlook, as demonstrated in previous research.^30,31^ Integrating human rater data alongside app‐based metrics enables calibration of automated tools against experiential benchmarks, improving both validity and user relevance.

### Contribution

This study aims to describe the SHM feature and evaluate its estimates of nature exposure through a user study, with the dual goal of refining the app and enhancing our understanding of how people rate natural environments. This study acts as a formative, route-based validation intended to quantify agreement between the apps estimates and human perception in a controlled set of challenging micro-environments and identify error patterns that motivate calibration/personalization. It is not intended as a population-level validation across diverse environments. Overall, this investigation contributes to the broader objective of scientifically informed app development. Specifically, the study is set to:

1. Present a methodology for app-based assessments of nature exposure.
2. Evaluate the app’s Green Index (GI), an estimate of the amount of nearby nature, through a user study.
3. Identify the level of individual variation in how people rate natural environments.
4. Explore possible individual and environmental factors that influence how people rate natural environments.

## Methods

### Sustainability and Health Motivator feature (SHM)

The SHM feature was built on top of an existing app created by *Kobla AS* (https://kobla.no/en/). The existing app was created to motivate users to engage in active transport, while the feature would add functionality to track and promote engagement with natural environments. To address the challenge of measuring time spent in different environments, the project developed the GI to assess the amount of nature in a location using mobile phones as measuring devices. The feature was designed to operate in background mode, requiring no user interaction to start measurements and no need for mounting or specific positioning of the phone. It was also required to have low resource costs in terms of memory usage, network bandwidth, and energy consumption. Privacy was a key consideration, meaning that no sensitive data should be shared with third parties. In addition, the solution needed to be phone-agnostic, providing consistent results across different phone models. Finally, it was designed to ensure a national scope, producing comparable results for all locations within Norway.

The standard smartphone nowadays has multiple features and sensors that could assist in determining if an environment is green The camera could be used for automated image-based detection of greenery. The microphone could capture sounds and noise patterns associated with green versus built environments, enabling classification and detection. Sensors such as the magnetometer, accelerometer, and temperature sensor could be used to distinguish between indoor and outdoor environments based on environmental and movement-related signals. Pulse and other health-related sensors could potentially be used to infer whether a person is in a more relaxed state. Finally, the global navigation satellite system (GNSS) could enable location tracking, where users’ positions may be shared with third-party services to obtain static or dynamic map information, as well as weather information for the user’s current geolocation. . The camera was excluded early in the developmental process due to the background-mode requirement. The microphone may have been useful, but since the phone’s placement is unknown (it could be in a bag, pocket, or hand), the sensor’s input would have required filtering. It was decided that the method that would give the most consistent results was using location data from the GNSS receiver and performing lookups in static maps available via the Norwegian Mapping Authority (Statens Kartverk) web service APIs. The mapping service offers multiple datasets for Norway. These maps are available in various formats and are based on satellite and plane photos, laser scans, property borders, and simulations. The maps are primarily delivered in two formats: vector or bitmap. Vector formats offer the advantage of richer detail and metadata for each map segment. In contrast, bitmap-based maps are pre-rendered on the server, significantly reducing computational load and battery usage on mobile devices. However, a limitation of bitmaps is that metadata, such as place names, are either embedded directly in the image or require additional bitmap layers to be represented. Given these considerations, it was decided to use bitmap-based maps to optimize performance on mobile devices in line with the requirements outlined above. These maps were used as the foundations for all app-based assessments.

#### Two datasets were selected as input for the GI

##### Topo

**Figure 1.**
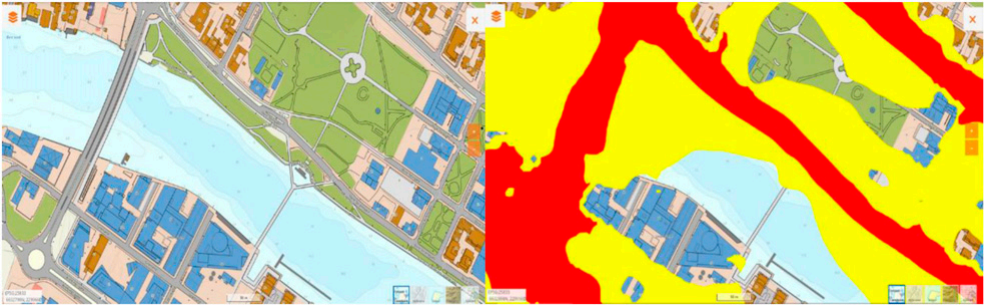
Screenshots of the maps used, showing the area used for this study. Left: Green = natural area. Light blue = water. Dark blue = building. Grey = roads, pavement, parking lots, etc. Pale pink = mixed peripheral spaces including residential gardens, entryways, urban open zones, etc. Right: Red = average daily traffic noise >65 dB. Yellow = average daily traffic noise 55-64 dB. Retreived from geonorge. no, av Geonorge, 2025, https://kartkatalog.geonorge.no/kart?lat=6633201.222009197&lon=229495.77191361735&zoom=11.515166308152821.

Topology map available on a web map tile server (WMTS) delivering images with a fixed size in different resolutions in PNG format. The advantage of the WMTS service is that images are cached on the server, providing fast response times for clients. These topology maps were used to identify different types of environments and elements, as shown in Table 1 and Figure 1.

**Table 1.** Overview of weights applied to different topographies and noise levels.

Map source	Map legend	Weight
Topography map	Building	0.0
	Water	1.0
	Green area	1.0
	Road	0.0
	Path	0.2
	Industrial	0.0
Noise map	≥65 dB	0.0
	55-64 dB	0.7
	0-54 dB	1.0

##### Noise

Noise maps are available as a Web Map Service (WMS) and deliver PNG images. The map has two noise thresholds based on the total number of vehicles using the road per 24 hours (Lden): red (Lden>65dB) and yellow (Lden>55dB). Traffic noise by itself may detract from a nature experience.^32^ Still, the noise maps were also used to differentiate between heavily and less trafficked roads as a proxy for urban activity in an area.^33^ Please refer to Table 1 and Figure 1.

The GI score for a given location is based on the concepts of GI_topo_ and GI_noise_. To calculate GI_topo_, different topographies in the topo map are weighted (0.1) based on how natural they are considered to be (Table 1). For a given location, a histogram of the different topographies within a 20-meter-radius circle around it is created. Metadata in the map (black/white) is classified as data-noise (noise-topo). The topographies are weighted and summed (sum-topology) as the total number of pixels minus topo-noise (total-noise), i.e.:

GI−topo=sum−topology/total−noise

Since the map’s topologies are graded by elevation (depth for water bodies), luminance must be extracted from the RGB (Red, Green, Blue) values in the PNG bitmap. This is done by converting RGB to a color space where luminance is a separate component. Luminance is then ignored when creating the histogram. GI_noise_ is based on available traffic-noise maps and is calculated from Lden (day-evening-night traffic-noise level) at specified measurement points. The map is colored with yellow and red noise zones. The noise zones are weighted and calculated in the same way as for GI-topo, but conversion to color space is not necessary (see Figure 1 for example screenshots of these maps). Finally, the GI was calculated using the following formula: mean GI_topo_ within a 20 m radius * mean GI_noise_ within a 20 m radius, which means that a GI score of 1 (or 100%) for a given location indicates an environment within a 20 meters radius consisting of 100% natural features without any traffic noise above 54 dB. To calculate the green index for a route, a mask of the route is created by drawing the route with a line thickness of 20 meters. The mask is applied to the map segment, and non-masked pixels are counted and weighted in the same way as for the location-based index.

The GI was integrated into the existing app. The app has a module for automatically recording users’ journeys, which were used as input to calculate the GI. The GI was then added to bonus rewards and other gameplay features in the app. The user interface was extended to include a GI indicator that displays the current GI at the user’s location. This allowed for both testing the GI and testing whether users can be nudged to spend more time in a green environment.

### Pilot testing

On the absence of a universal reference standard for GI parameter choices. There is no single, established ‘gold standard’ against which to validate spatial radius, topography weightings, noise weighting, or combination rules for app-based indices of nearby nature. To produce an initial GI configuration, we used an expert-informed, heuristic approach. Pilot assessments were conducted collaboratively by the app developer and a multidisciplinary scientific team with expertise in health-enhancing physical activity, nature-based activities, and environmental psychology. These sessions compared app outputs to expert judgments. These pilot assessments involved repeated field-based evaluations across a range of environmental contexts, including clearly urban, clearly natural and mixed environments, allowing the team to iteratively assess and refine the weights, thresholds, and spatial parameters used to calculate the green index (GI). This iterative process aimed to align algorithmic outputs with expert judgments of environmental naturalness and to establish the GI configuration used in the subsequent user study. The weights reported in Table 1 (e.g., Path = 0.2, Water = 1.0) reflect these expert-informed choices. They were selected to (a) reflect a judgment that water and contiguous green cover contribute strongly to perceived naturalness, (b) reduce the influence of thin linear features (paths) relative to extensive green/blue patches, and (c) de-emphasize built/industrial topographies and highly trafficked areas. These weights were the foundation for calculating GI-topo. GI_topo was combined with GI_noise by multiplying the mean values within a 20 m radius to reflect the assumption that the restorative/natural experience at a given location depends jointly on the presence of natural features and the absence (or low level) of traffic noise/urban activity, e.g., high naturalness with high noise should reduce the net GI. The 20 m radius was chosen to approximate the immediate surroundings that a pedestrian would typically perceive. These choices were made by the pilot field sessions and by the app’s design constraints (background operation, low energy and data usage. We emphasize that design choices are initial, expert-informed heuristics rather than validated measurement parameters; their empirical calibration is an explicit aim of further work.

During the pilot phase, a high degree of agreement was observed between the app-generated GI scores and expert ratings in environments that were clearly characterized as either highly natural or highly urban. In contrast, environments comprising mixed or transitional landscapes, such as areas with interspersed built and natural elements, elicited greater variability in expert assessments and more frequent discrepancies with app-based estimates. These complex settings highlighted the challenges of characterizing nature exposure in heterogeneous urban environments and underscored the need for more systematic and structured evaluation through a user study involving a broader range of raters and locations.

### User study

#### Recruitment and study design

The user study was designed as a within-subjects quasi-experimental validation study conducted to identify specific issues to be addressed in further adjustments of the GI. Participants were recruited via advertisements on the university’s social media accounts, local radio broadcasts and news outlets, our professional network, and word of mouth. Participants were included if they were able to complete a 20-minute walk. Since the study was exploratory in nature and not designed as a definitive population-level validation. We therefore did not perform a formal a priori sample-size calculation. Instead, we recruited a convenience sample of 30 participants (one participant was excluded from analysis for invalid responding, leaving n = 29), consistent with prior user-testing and small-scale validation studies.^34^ The study was conducted in line with the Guidelines for Research Ethics in the Social Sciences and the Humanities given by the Norwegian National Committee for Research Ethics in the Social Sciences and the Humanities. The study also complied with national regulations regarding data protection and was approved by the Norwegian Agency for Shared Services in Education and Research (Sikt; Ref. 131488). All participants provided written informed consent before participation in the study.

#### Procedures

**Figure 2.**
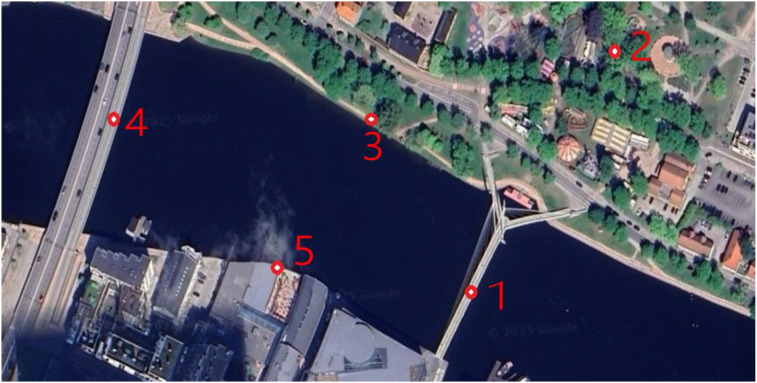
Satellite photo of the area used in the experiment, with each location marked by a red circle. Location 1 = right center, in the middle of a pedestrian bridge. Location 2 = top right, in a park. Location 3 = top center, in a green area alongside the river. Location 4 = left center, on top of a trafficked bridge. Location 5 = bottom center, in a built area alongside the river.

**Figure 3.**
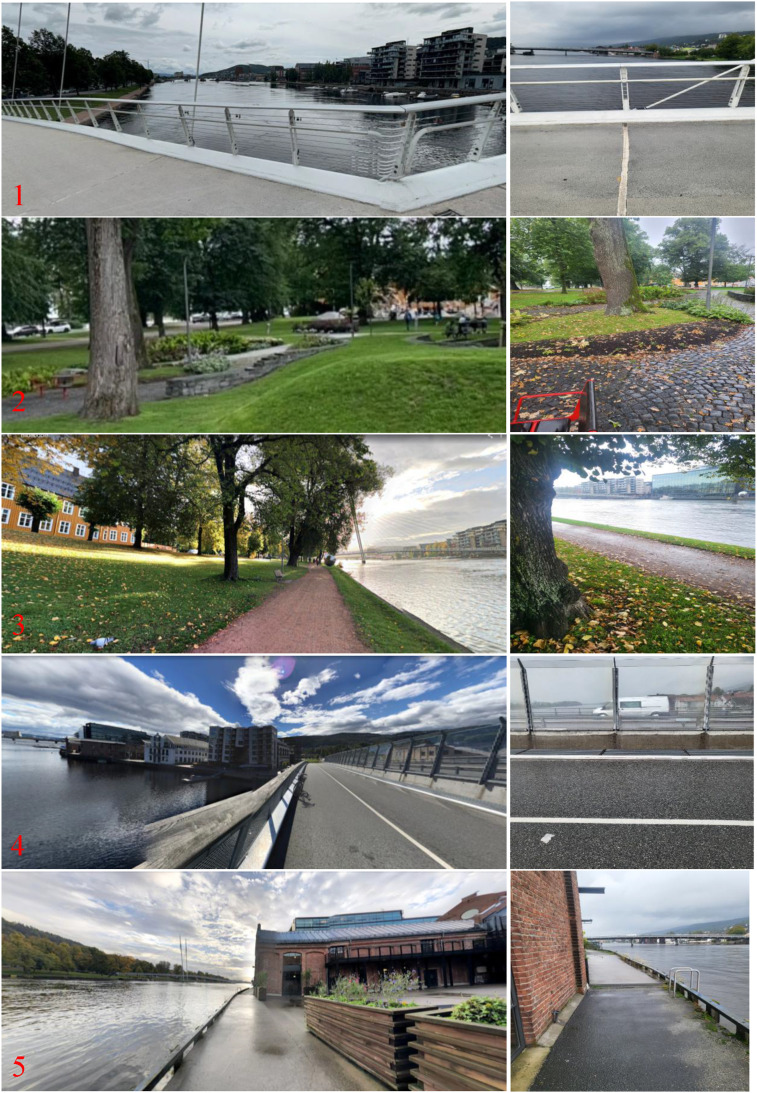
Photos of each location, in representative weather conditions for the time of experiments, in order 1-5 from top to bottom.

Based on the pilot study’s learnings, five locations were identified for the study (Figures 2 and 3). These locations, which were connected by a walking route and contained varying degrees and types of natural and built elements, were chosen because they represented different challenges for the GI. Specifically:

- Location 1 (Pedestrian bridge): outside the traffic noise area, halfway across a pedestrian bridge over a large river, with a view of buildings and green landscapes (nearby parks and forest-covered hills in the distance);
- Location 2 (Park): outside the traffic noise area, in the middle of an urban park, with a view of park elements and built elements (a pavilion and a nearby street with buildings);
- Location 3 (River, green): within the yellow (55-64 dB) traffic noise area, on an unpaved trail in a large patch of grass and trees along the riverside, with a view of a large bridge and buildings on the opposite riverside.
- Location 4 (Trafficked bridge): within the red (>65 dB) traffic noise area, halfway across a trafficked bridge supporting a major road, with a view of buildings and green landscapes (parks and forest-covered hills in the distance).
- Location 5 (River, grey): outside the traffic noise area, on a paved sidewalk along the river and a line of buildings, with a view of green landscapes (parks and forest-covered hills on the opposite riverside).

Participants were asked to walk along this route and stop at each location to allow a within-subjects design, increasing internal validity, as participants served as their own controls.

The researchers would greet the participants at the main entrance of the university. Before initiating the experiment, participants filled in a brief survey assessing background information (age, gender, and nature connectedness). Subsequently, they walked to the study route, including all five locations, which took about 20 minutes (excluding time spent answering the questionnaire). The researcher walked directly behind the participant (∼10 m), in line with previous research. The walking direction was randomized: half of the participants walked from location 1 to 5, while the other half walked from location 5 to 1 to test for potential effects of walking direction. Participants stopped at each of the five different locations and were asked to complete a brief survey, on a specific smartphone provided by the researcher, assessing “perceived nature rating”, perceived environmental restorativeness, and overall feelings (see Instruments). When the participants returned to the starting point, a brief unstructured interview was conducted to gain a more in-depth understanding of participants’ experience of the quality of the natural environments. The study was conducted between September 23^rd^ and September 30^th^ in the city of Drammen, Norway, during a period of stable, semi-cloudy weather, and none of the walks were conducted in rainy, windy, or sunny conditions to limit the impact of weather on participants’ assessments of the natural environments.

#### Instruments

##### Perceived Nature Rating (PNR)

This measure consisted of the mean score of two questions: “How natural do the surroundings appear to you?” (item 1) and “Assess the amount of natural elements in the surroundings” (item 2). The first question assessed participants’ subjective experience, considering all elements that may influence it, such as the types of natural elements and their aesthetics. The second question was intended to elicit a more objective evaluation of the area, similar to the app’s GI assessment. Both questions were rated on a scale from 0 to 100 % to align with the GI. The Cronbach’s alphas for these two questions ranged from 0.77 to 0.91 across the five locations, indicating a high level of agreement. However, since these two questions are conceptually different, they were also considered individually in the analysis, in addition to the combined PNR.

##### Perceived Environmental Restorativeness (PER)

The perceived environmental restorativeness scale^35,36^ assesses the overall restorative potential of environments and was used in this study as an indicator for the health-enhancing potential of the different locations. The scale consists of 16 items assessing the subjective evaluation of four environmental qualities according to the Attention-restoration theory^37,38^: i) fascination (five items, e.g., “The setting has fascinating qualities”), being away (two items, e.g., “It was an escape experience”), coherence (four items, e.g., “There is too much going on”), and compatibility (five items, e.g., “have a sense that I belong there”). Items for coherence are negatively phrased, such that lower scores indicate better coherence. For ease of interpretation of results, we reversed the scoring so that higher values reflect greater coherence.

##### The Feeling Scale (FS)

The feeling scale is a measure of a person’s overall emotional state,^39^ and it was administered at each location as another indicator of the acute benefits to health and well-being of each location. It consists of a single item inquiring “How are you feeling right now?”, rated on an 11-point bipolar scale measure ranging from -5 (“very bad” to +5 (“very good”).

##### Perceived weather conditions

At the final location, participants were asked to rate the weather on a scale from 1 to 10, with anchors 1 = terrible, 5 = moderate, and 10 = fantastic, immediately upon completing the walk. This single retrospective rating may reflect momentary mood in addition to actual weather conditions and is therefore analyzed descriptively and in exploratory correlations with the primary outcomes.

##### Background characteristics

Participants’ gender, age, and levels of nature connectedness were collected before the walk to describe the study sample and assess associations with the outcome variables. The participants’ baseline level of nature connectedness (i.e., a person’s cognitive and affective attachment to the natural world) was assessed by the trait version of the Connectedness to Nature scale (Mayer & Frantz, 2004)^40^. The scale consists of 14 items (e.g., “I often feel like I am part of the web of life”), each rated on a five-point Likert scale. Additionally, the time of day was recorded by the service used to administer the survey (Nettskjema, University of Oslo, Oslo).

##### Unstructured interviews

After completing the tour, the examiner asked participants to share their perceptions of the different locations and why they rated them as they did. The main points emerging were noted to provide further insight into the quantitative data.

### Analysis

The Shapiro-Wilk test was used to assess the assumption of normality, and none of the outcomes were significantly different from normality. Data were further explored for missing values and possible outliers –none were identified. The assumption of sphericity was assessed by Mauchly’s test of sphericity. A mixed between-within subjects analysis of variance (ANOVA) was performed for all the primary outcomes (PNR, FS, and the four components of PER) using the five locations as repeated measures (within-subjects factor) and the two dichotomous outcome variables, gender and walking direction, as between-subjects factors. If significance was achieved in the within-subjects test, post hoc pairwise comparisons with Bonferroni’s correction of alpha were conducted among all possible combinations of locations. The purpose was *i*) to establish possible differences in the measured outcomes among the different locations and *ii*) to assess potential effects of gender and walking direction on the outcome variables. To further explore potential influences of other factors, Spearman’s rank correlation coefficient was used to assess potential associations between the outcomes and the other numeric variables (Age, Nature connectedness, Time of day, and Perceived weather). To reduce the number of statistical comparisons and limit the risk of type I error, the mean across all five locations for the outcome variables was used in the correlation analysis. Effect sizes (r_s_) were interpreted according to Cohen’s recommendations: 0.1 = small, 0.3 = medium, and 0.5 = large. In comparison, descriptive statistics are presented for each of the five locations. Data are presented as mean and standard deviation (M ± SD) and ranges. Differences between the GI assessments and the mean PNR at each location were also calculated. The statistical analysis was performed in IBM SPSS statistics 29 (IBM., New York). The significance level was set at p < 0.05.

## Results

One participant was removed from the analysis due to consistently selecting the top option (strongly agree, 100%, etc.) in all locations for both positively and negatively phrased questions. Thus, the final sample consisted of 29 participants, 14 men and 15 women. The mean age of participants was 39.6 ± 16.1 (range 18-74) years, and they reported a baseline mean level of nature connectedness of 3.62 ± 0.46.

**Figure 4.**
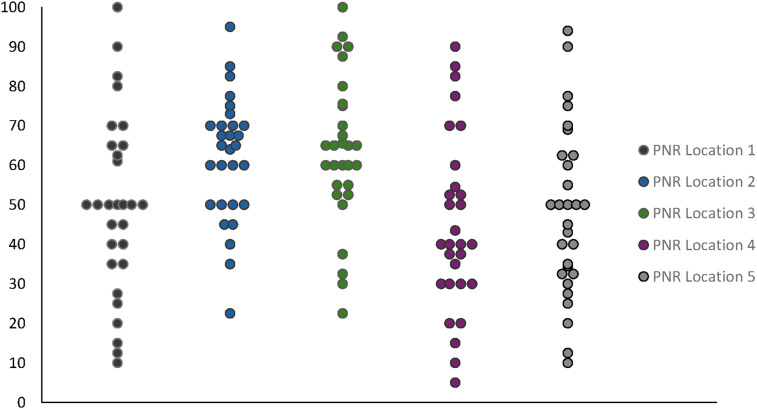
Scatterplot of each individual rating of perceived nature rating (PNR) at each location.

Ratings at each location are presented in Table 1. The difference between the GI and the mean PNR at each location was +38.1, +1.2, -32.5, -23.8, and +2.0 (values for locations 1 to 5, respectively). The difference between the highest and lowest GI was 67 percentage points, while the difference between the highest and lowest PNR was 18.7 percentage points. Considerable individual variation was observed across all locations and assessments, as illustrated by the standard deviations in Table 2 and the scatterplot in Figure 4.

**Table 2.** Ratings at each location for the different outcome variables presented as means ± standard deviation.

Measure	Location 1	Location 2	Location 3	Location 4	Location 5
GI (%)	88	63	31	21	50
PNR (0-100)	49.9 ± 22.8^3^	61.8 ± 15.7^4,5^	63.5 ± 18.7^1,4,5^	44.8 ± 22.4^2,3^	48.0 ± 21.4^2,3^
Appear natural	51.6 ± 26.4	60.1 ± 19.2^4^	62.6 ± 20.4^4,5^	44.8 ± 25.3^2,3^	48.0 ± 24.8^3^
Amount natural	48.2 ± 21.8^2,3^	63.5 ± 15.4^1,4,5^	64.4 ± 18.9^1,4,5^	44.7 ± 21.3^2,3^	48.0 ± 19.7^2,3^
Being away	5.8 ± 2.8	6.2 ± 2.2	7.1 ± 2.3^4,5^	4.8 ± 2.8^3^	5.4 ± 2.6^3^
Fascination	6.3 ± 2.1	6.1 ± 1.5	6.9 ± 1.7^4,5^	4.9 ± 2.4^3^	5.6 ± 2.1^3^
Coherence	5.6 ± 2.4	5.9 ± 2.8	6.9 ± 2.3^4^	4.7 ± 2.1^3,5^	6.3 ± 2.3^4^
Compatibility	5.8 ± 2.3^3^	6.2 ± 1.7^4^	7.2 ± 1.7^1,4,5^	4.5 ± 2.4^2,3^	5.6 ± 2.2^3^
FS	2.5 ± 1.8	2.4 ± 1.7	3.2 ± 1.6^4,5^	1.9 ± 1.8^3^	2.1 ± 2.0^3^

GI = green index; PNR = perceived nature rating; Appear natural = item 1 of the PNR; Amount natural = item 2 of the PNR; FS = Feeling scale. Superscript numbers indicate statistically significant differences emerging from the post-hoc analysis:

^1^significantly different from location 1 (Pedestrian bridge).

^2^significantly different from location 2 (Park).

^3^significantly different from location 3 (River, green).

^4^significantly different from location 4 (Trafficked bridge).

^5^significantly different from location 5 (River grey).

There was a significant main effect of location for all outcomes: PNR F (4,100) = 10.71, p < 0.001; Being away F (4,100) = 5.17, p < 0.001; Fascination F (4,100) = 7.55, p < 0.001; Coherence F (4,100) = 6.78, p < 0.001; Compatibility F (4,100) = 9.05, p < 0.001; FS F (4,100) = 5.96, p < 0.001. The directions of magnitudes of these effects across locations are displayed in Table 2. Generally, significantly higher scores were reported for Location 3 (River, green) compared to Location 4 (Trafficked bridge); considerably higher scores were also reported for Location 3 (River, green) compared to Location 5 (River, grey), except for coherence; otherwise, some sporadic significant differences were observed between specific Locations (please refer to Table 2 for details). The two items of PNR followed the same overall pattern, but some minor differences in mean values and lower standard deviations resulted for item 2 (amount of natural elements) compared to item 1 (how natural the surroundings appear), resulting in more locations with significant differences for item 1 (please refer to Table 2 for details).

The repeated measures ANOVA revealed a significant interaction between walking direction and gender for PER component coherence (see Table 3). Pairwise comparisons indicated that differences drove this effect at locations 2 and 3, where male participants rated the environment as significantly more coherent compared to female participants (Location 2: 7.0 ± 2.1 vs 4.2 ± 3.1, p = 0.013; Location 3: 7.9 ± 1.8 vs 5.3 ± 2.8, p = 0.028). No other significant interaction effects emerged from the repeated measures ANOVA.

**Table 3.** Interaction effects between the outcome variables across all five locations and gender and walking direction, respectively.

Measure	Male	Female	Gender	Clockwise	Counterclockwise	Walking direction
PNR	54.1 ± 18.1	53.1 ± 17.3	F (4,100) = 0.70, p = 0.594	53.3 ± 19.0	53.9 ± 16.3	F (4,100) = 1.15, p = 0.337
Appear natural	53.7 ± 20.1	53.1 ± 18.3	F (4,100) = 1.02, p = 0.400	53.4 ± 20.9	53.4 ± 17.4	F (4,100) = 0.53, p = 0.716
Amount natural	54.5 ± 16.9	53.1 ± 17.0	F (4,100) = 0.27, p = 0.899	53.0 ± 18.1	54.4 ± 15.9	F (4,100) = 1,74, p = 0.147
Being away	5.5 ± 1.6	6.2 ± 2.1	F (4,100) = 1.01, p = 0.371	5.9 ± 1.9	5.8 ± 1.9	F (4,100) = 2.17, p = 0.077
Fascination	5.9 ± 1.3	5.9 ± 1.6	F (4,100) = 1.38, p = 0.246	6.0 ± 1.4	5.9 ± 1.6	F (4,100) = 1.83, p = 0.129
Coherence	3.9 ± 1.3	4.3 ± 2.3	F (4,100) = 4.58, p = 0.002	4.5 ± 1.6	3.8 ± 2.1	F (4,100) = 1.90, p = 0.116
Compatibility	5.9 ± 1.3	5.7 ± 1.5	F (4,100) = 0.94, p = 0.442	6.0 ± 1.3	5.7 ± 1.4	F (4,100) = 1.39, p = 0.243
FS	2.5 ± 1.3	2.4 ± 1.6	F (4,100) = 0.64, p = 0.631	2.3 ± 1.3	2.5 ± 1.6	F (4,100) = 1.21, p = 0.313

PNR = perceived nature rating; Appear natural = item 1 of the PNR; Amount natural = item 2 of the PNR; FS = Feeling scale.

The correlation analysis is displayed in Table 4 as a correlation matrix. The matrix shows several significant medium to large correlations, some of which are particularly relevant. PNR showed moderate to high positive correlations with FS and the most effective components, except for coherence. Perceived weather was also positively associated with FS and all PER components except coherence, again showing moderate to high effect sizes. Time of day was negatively associated with being away, whereas nature connectedness was positively related to the FS. No other statistically significant correlations were observed.

**Table 4.** Displays a correlation matrix to assess potential associations between variables.

Measure	Age	NCS	Time of day	Weather	PNR	Appear natural	Amount natural	Being away	Fasci-nation	Coher-ence	Compati-bility	Feeling scale
Age	1	​	​	​	​	​	​	​	​	​	​	​
NCS	0.147	1	​	​	​	​	​	​	​	​	​	​
Time of day	0.265	0.180	1	​	​	​	​	​	​	​	​	​
Weather	0.091	0.158	-0.247	1	​	​	​	​	​	​	​	​
PNR	0.194	-0.059	-0.101	0.536**	1	​	​	​	​	​	​	​
Appear natural	0.172	-0.091	-0.107	0.544**	0.974**	1	​	​	​	​	​	​
Amount natural	0.214	-0.031	0.004	0.476**	0.953**	0.883**	1	​	​	​	​	​
Being away	0.147	-0.052	-0.381*	0.474**	0.510**	0.519**	0.493**	1	​	​	​	​
Fascination	0.110	0.030	-0.229	0.637**	0.512**	0.544**	0.445**	0.705**	1	​	​	​
Coherence	0.358	0.013	-0.165	0.050	0.113	0.032	0.167	0.029	0.003	1	​	​
Compatibility	0.103	-0.019	-0.276	0.593**	0.609**	0.579**	0.590**	0.748**	0.721**	-0.059	1	​
FS	0.257	0.369*	-0.173	0.640**	0.574**	0.532**	0.583**	0.414**	0.683**	-0.239	0.593**	1

All significant outcomes are indicated in bold. NCS = nature connectedness scale; PNR = perceived nature rating; FS = Feeling scale. * p < 0.05; ** p < 0.01.

The main and recurring themes emerging from the unstructured interviews were a dichotomy in people’s perceptions of the types of natural environment and traffic noise. Some participants reported a clear preference for green spaces compared to blue, while others reported the opposite, e.g., “you know, I preferred the park, it is more similar to a forest, which is the real nature” and “I grew up along the sea, and that is what nature is to me”. These findings were corroborated by quantitative data showing that 8 participants reported higher PNR for location 1 (Pedestrian bridge) than for location 2 (Park), while 17 reported the opposite. Four reported equal PNR in both places. Regarding traffic noise, some participants were adamant that it had a significant negative impact on their experience, e.g., “the traffic noise ruined the experience”. In contrast, others reported little impact from these factors on their perception of the area, e.g., “I did not notice the traffic noise”. Accordingly, 17 participants reported higher PNR for location 1 (Pedestrian bridge) while 12 reported no difference or higher values for location 4 (Trafficked bridge).

## Discussion

**Figure 5.**
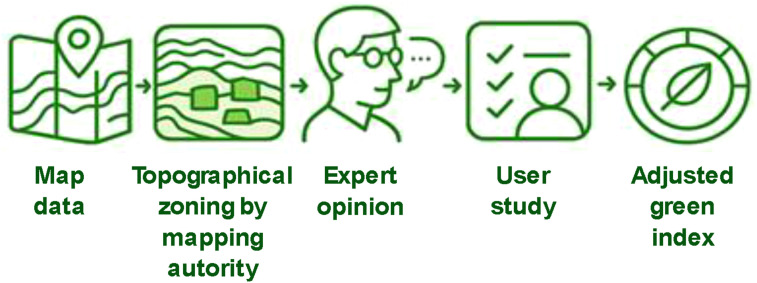
Schematic representation of how each step in the developmental process is layered on top of each other to create an accurate and functional version of the green index with real-world application.

SHM is a novel app feature that aims to track time spent in nature to promote health and wellbeing by encouraging outdoor activity. To achieve this, the SHM calculates the GI using topographical map-data. The developmental process aimed at adding sequential layers of complexity to the GI, improving its accuracy and usability, as shown in Figure 5. Specifically, the app’s requirements, the maps and topographical zoning provided by the mapping authority, and the pilot study that included experts in the field laid the foundation for the GI used in this study. Through the latest reiterative step (i.e., the user study), , suggestions can be made for further changes to the app, which may be beneficial to accomplish the goal of promoting health and sustainability through engagement with natural environments.

The results of this user study revealed several aspects that may guide the further development of the SHM app. Each of these aspects is outlined below with suggestions for how they may be incorporated in the developmental process.

Most importantly, significant discrepancies between the GI and PNR of the five locations were observed. This highlights fundamental differences between computer evaluations and human perceptions of the natural environment and suggests that modifications to the GI are warranted. For instance, while a bridge in the middle of the river (i.e., Location 1 and 4) may objectively indicate a highly natural area, factors such as cultural background, lived experience, and perceived novelty of an environment may influence individual ratings.^20,23^

In general, the GI showed greater distinctions among the five locations, while human participants tended to assign more homogeneous values (Table 2). This may reflect differences between algorithmic and human raters for each specific location, or that human raters are influenced by carry-over effects from previous locations. Regardless, the health-related indicators (PER and FS) were both moderately to highly correlated with PNR. Consistent with this association, the findings in Table 2 show that PER and FS| align more closely with PNR than with GI. For example, location 3 (River, green) ranked as the most “natural” location according to PNR and simultaneously had the highest ratings of PER and FS. Given that higher subjective ratings for PER and FS are associated with improved psychological and physiological restoration in the literature,^35–39^ these findings suggest that Location 3 (River, green) may offer greater restorative benefits. Yet the GI placed location 3 (River, green) second-to-last among all locations and underestimates the location by 32.5 points compared to participants’ evaluations. Similarly, the GI overestimates location 1 (Pedestrian bridge) by 38.1 points compared to PNR. Practically, these discrepancies indicate caution when relying on GI for site selection or design decisions. Combining GI with direct participant ratings (PNR, PER, FS) should yield a more complete and reliable assessment, for example by reducing the difference in the GI between different natural locations.

Large inter-individual variability was observed among participants in their ratings of the locations (both for PNR and PER). Previous studies have shown some degree of variability when participants are presented with images of nature (e.g. Menser et al., 2021)^41^, but not to the degree found in the present study when immersed in real-world environments. The level of variability observed underscores the subjective nature of environmental perception, where personal experiences, cultural backgrounds, and individual preferences, etc., play crucial roles.^8,17–21,23^ This variability poses a challenge for creating universally applicable assessment tools, as users may discredit information that does not align with their personal evaluations.^24^ One approach to tackle this issue could be to add a level of personalization and individualization to the GI. In our study, we included assessments of age, gender, and baseline level of nature connectedness, as they have previously been reported as moderators of the health outcomes of nature exposure.^42–44^ However, our results did not show associations between these baseline factors and any of the outcome variables, except for nature connectedness and FS, suggesting that these factors may not be appropriate for personalization. Other factors have previously been proposed as individual moderators of the effects of nature exposure, such as the type of motivation for visiting natural environments,^19^ ethnicity/culture,^8,20^ susceptibility to stress,^21^ and the level of attention directed towards natural elements in an area.^22^ Factors such as these may be used to address the issue of individual differences, but the effects remain to be tested for app-based assessments of nature exposure. Another approach to solve the problem of considerable individuation variation in the evaluations of specific locations could be to limit the level of detailed statistics reported to the user, e.g., only presenting summary statistics of how many minutes spent in nature or within a specific range of GI across a day or a week to mask any significant disagreements between the user and GI to particular locations. The app would still achieve its purpose by nudging the user to increase its daily/weekly averages.

The issue of presenting detailed statistics, or not, also relates to the intensity of the “nature dose”. The concept of nature does commonly refer to intensity (i.e., quantity and quality) and duration^45^ of a human-nature interaction. Previous research has shown that even low-intensity nature experiences, such as viewing nature through a home window, can induce psychological benefits.^45^ In line with this assumption, current recommendations largely advise people to seek time in nature, independent of intensity, and instead focus on duration^8^ and access.^6^ Considering this, it is pertinent to suggest that the information presented by nature-tracking apps to participants could be reduced to a dichotomous threshold (i.e., “in nature” vs. “not in nature”) rather than continuous estimates (i.e., percentage scores). Given that even low-intensity environments are beneficial,^45^ it may be argued that the cut-off for a dichotomous threshold should be set relatively low. For instance, using GI ≥ 20% as a threshold for sufficient presence of nature would still allow for capturing environments with reasonably high levels of urban nature, which were associated with relatively high levels of PNR in this study (e.g., location 4). Moreover, such an approach may promote human-nature interactions even in environments with relatively low levels of nature (e.g., urban settings), whilst also contributing to greater awareness among people of the presence of nature in their living environments. For instance, in a systematic review investigating ways to foster nature connectedness in the adult population, Sheffield et al. (2022) recommend campaigns that raise awareness of everyday nature and support the development of personal practices involving regular contact with nature. However, some research suggests that higher natural intensity is more beneficial to human health than low-intensity environments.^46^ This is supported by the significantly higher scores for PER and FS assigned to Location 3 (River, green) compared to Location 4 (Trafficked bridge) and suggests that the app should provide some user feedback regarding the nature intensity of an environment, e.g., by using a trichotomous approach by dividing environments into no nature, sufficient nature (e.g., GI ≥ 20%), and abundant nature. Considering the misalignment between the objective (GI) and subjective (PNR, PER, and FS) assessments for Location 3 (River, green), specifically, this study needs to refine the GI to identify a threshold for environments considered abundant in nature. One similar app has adopted this threshold-based approach and divided natural interactions into five levels.^3^ Still, there are some caveats to this approach. Firstly, as demonstrated in this study, objective assessments based on geo-localized data may not be able to distinguish nuances in the quality of natural environments. Secondly, it has been theorized that the associations between the intensity of nature may be non-linear.^45^ For instance, specific natural environments with reduced visibility of the broader landscape (i.e., low prospect, such as forests with thick vegetation) may be perceived as less restorative than environments with high prospect,^47^ while natural environments that incorporate some built elements are optimal for stress reduction.^48^ This further underscore the challenges of quantifying human-nature interactions with objective data alone. With no optimal solution in sight, it is essential to consider the app’s purpose when deciding on the level of detail to present to the user. The purpose of the SHM feature is to promote health and sustainability, which suggests it should be able to identify and foster highly natural environments with both “sufficient” (Location 4) and “abundant” (Location 3) nature. Regardless of the chosen approach, it would require a GI capable of balancing the different elements used in the algorithm to provide an accurate categorization of environments, such as assigning appropriate weights to traffic noise.

In our assessment, traffic noise, which varied widely across the five locations, served as an indicator of potential stressors detracting from the nature experience, yet the app’s interpretation of this noise significantly diverged from participants’ perceptions. While traffic noise may be a valuable proxy for urban activity, it did not substantially affect participants’ evaluations of the environment. Both quantitative and qualitative outcomes indicated that many participants found traffic and traffic noise had little to no impact on their assessment of the area. At the same time, a minority reported that traffic and traffic noise had a larger, meaningful impact on their ratings. The divergence between the app and human raters may stem from the fact that humans integrate the sound and visual impressions of an area in a more complex way than an algorithm.^49^ Since the majority found that traffic noise had little impact on their ratings, it may be argued that this factor could be reduced or even omitted from the GI calculation. However, previous research has shown that walking along urban roads reduces the positive mood effects of nature exposure^33^ and that natural sounds have a positive impact on human health, while urban sounds have a negative effect,^32,49^ suggesting that the inclusion of traffic noise in the apps algorithms is warranted despite the divergence in participants responses in our study. Further refinement of the app’s integration of traffic noise as a proxy for urban activity may help address some of these issues, such as reducing its impact on the GI, but excluding this factor from the evaluation is probably counterproductive given the established adverse health effects of urban sounds.

The correlation analysis further revealed an association between self-rated weather conditions and the outcome variables PNR, perceived environmental restorativeness, and the feeling scale, equivalent. to medium to large effect sizes. Perceived weather conditions were the only factor most consistently associated with other outcomes. Previous research has indeed shown that people’s general feelings during physical activity, including nature visits, can affect future motivation.^50^ Hence, based on the findings of the present study and previous research showing that weather can influence mood and affect in general,^51^ it is pertinent to propose adjustments to the GI that personalize cues and recommendations based on users’ weather preferences or tolerances. At the same time, caution is advised when interpreting these results. Responses are likely influenced by mood-congruent judgment as well, i.e., the tendency to give more positive scores when in a good mood (Mayer et al., 1992). It may be that people who enjoyed the experiment or were in a generally good mood that day tended to give higher scores to perceived weather, PNR, PER, and FS. Thus, further research is warranted to understand the influence of both objective and perceived weather conditions on people’s perception of nature, as well as the extent to which this factor should be included in app-based assessments (Mayer et al., 1992).^52^

Our study has some weaknesses that should be taken into consideration. The main aim of the study was to evaluate the green index through a user study. This was done using a convenience sample of 29 participants across five locations. These five locations were selected based on pilot testing that showed these environments were challenging to rate by both the app and experts. The rather low number of participants and number of test locations limits the potential to extrapolate the findings to the general population and other locations; e.g., pilot testing also showed a high level of agreement among raters and the app across highly natural and highly urban environments. Thus, this user study should be considered part of an app’s developmental process to adjust the GI, rather than a study aiming to provide accurate data at the population level. Larger-scale studies across population groups and geographical areas are the next logical steps after this first user evaluation of the GI. The number of participants was also relatively low for statistical analysis, and small but meaningful associations may have been overlooked because no statistically significant effects were observed in our study. Further research within this field should aim to conduct large-scale investigations of the level of convergence between algorithmic and human ratings of natural environments. This could be done when the SHM app feature is ready for the market and open to the public.

## Conclusion

This formative evaluation shows that app-based assessments of natural environments can diverge broadly from users’ subjective evaluations. Moreover, users’ subjective evaluations tend to be more strongly associated with psychological indicators, with potential implications for both the app’s persuasive value and its overall health-promoting outcomes. At the same time, the considerable inter-individual variation in participants’ ratings illustrates how human perceptions and interpretations of “nature” are widely nuanced. For instance, participants in this study showed differences in how they rate green vs. blue areas, sensitivity to traffic noise and urban activity, and weather conditions. Altogether, this suggests that app-based assessments of the natural environment, such as the GI, should be calibrated against individualized subjective ratings from the users. The observed inter-individuality further warrants careful design of user feedback, such as prioritizing aggregated summary statistics over detailed information. These insights should guide the refinement of health‐oriented nature apps to balance rigorous models with human‐centered feedback.

## Data Availability

The datasets generated during and/or analyzed during the current study are available from the corresponding author on request.*
